# Prediction of fluid responsiveness in intensive care (PREFERENCE study): fluid challenge versus passive leg raising in high-risk surgical patients

**DOI:** 10.1186/cc10846

**Published:** 2012-03-20

**Authors:** M Cecconi, F Caliandro, J Mellinghoff, D Dawson, S Ranjan, M Hamilton, M Grounds, A Rhodes

**Affiliations:** 1 St Georges Hospital, London, UK

## Introduction

The aim of this study is to evaluate whether the passive leg raising (PLR) maneuver could be used to predict fluid responsiveness in awake postoperative patients admitted to the ICU. PLR has been demonstrated to be a good indicator of fluid responsiveness even in spontaneously breathing patients, but few data are available in the immediate postoperative period. Nexfin is a new cardiac output monitor that measures and tracks stroke volume (SV) by analyzing the arterial pressure pulse contour noninvasively from a finger probe.

## Methods

We enrolled self-ventilating patients admitted to the ICU postoperatively. A PLR maneuver (45° bed tilt from the 30 to 45° head up) was performed and followed by a fluid challenge (FC, 250 ml fluid bolus over 5 minutes). Changes in SV during PLR and after administration of FC were monitored with the Nexfin monitor. Receiver operator characteristic (ROC) analysis was performed.

## Results

**Figure 1:**
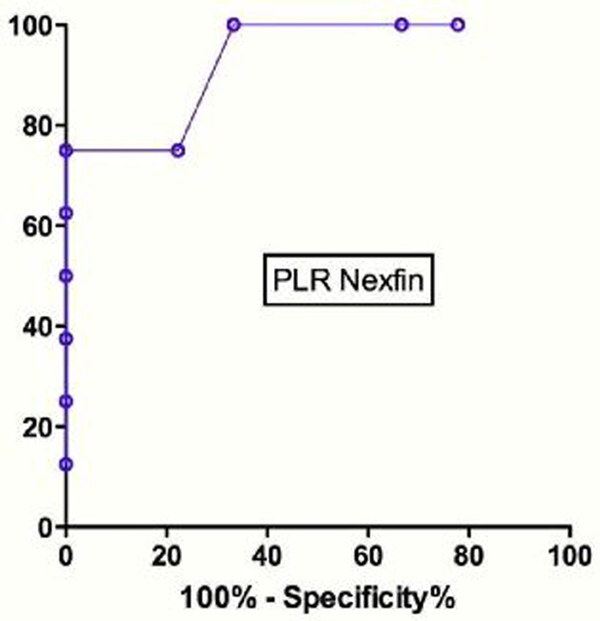


Forty-five patients were enrolled. Twenty-three patients responded to the FC with an increase of SV >5%. Twenty-eight patients (62%) were excluded from the PLR analysis as a result of haemodynamic instability (difference in heart rate, mean arterial pressure or SV baselines pre PLR and pre FC >5%). Seventeen patients were analyzed. The area under the curve for the ROC analysis was 0.93 (SE = 0.06; *P *= 0.003) (Figure 1). A SV increase >1% during a PLR test predicts a SV increase >5% after FC with a sensitivity of 75% and a specificity of 78%.

## Conclusion

In 62% of patients a PLR test could not be performed due to haemodynamic instability. In these patients, FC is the best way to assess fluid responsiveness. During haemodynamic stability PLR shows great sensitivity and specificity to predict fluid responsiveness. The Nexfin monitor can be used to track SV changes both during FC and during a PLR test.

